# Apelin/APJ signaling suppresses the pressure ulcer formation in cutaneous ischemia-reperfusion injury mouse model

**DOI:** 10.1038/s41598-020-58452-2

**Published:** 2020-01-28

**Authors:** Sahori Yamazaki, Akiko Sekiguchi, Akihiko Uchiyama, Chisako Fujiwara, Yuta Inoue, Yoko Yokoyama, Sachiko Ogino, Ryoko Torii, Mari Hosoi, Ryoko Akai, Takao Iwawaki, Osamu Ishikawa, Sei-ichiro Motegi

**Affiliations:** 1 Department of Dermatology, Gunma University Graduate School of Medicine, Maebashi, Japan; 2 Division of Cell Medicine, Department of Life Science, Medical Research Institute, Kanazawa Medical University, Ishikawa, Japan

**Keywords:** Skin diseases, Immunopathogenesis

## Abstract

Several studies have demonstrated potential roles for apelin/APJ signaling in the regulation of oxidative stress associated with ischemia-reperfusion (I/R) injury in several organs. Objective was to assess the role of apelin/APJ signaling in the development of pressure ulcers (PUs) formation after cutaneous I/R injury in mice. We identified that cutaneous I/R injury increased the expression of apelin in the skin at I/R site. Administration of apelin significantly inhibited the formation of PUs. The reductions of blood vessels, hypoxic area and apoptosis in I/R site were inhibited by apelin injection. Oxidative stress signals in OKD48 mice and the expressions of oxidative stress related genes in the skin were suppressed by apelin injection. H_2_O_2_-induced intracellular ROS and apoptosis in endothelial cells and fibroblasts were suppressed by apelin *in vitro*. Furthermore, MM07, biased agonist of APJ, also significantly suppressed the development of PUs after cutaneous I/R, and the inhibitory effect of MM07 on PUs formation was higher than that in apelin. We conclude that apelin/APJ signaling may inhibit cutaneous I/R injury-induced PUs formation by protecting the reduction of vascularity and tissue damage via suppression of oxidative stress. Exogenous application of apelin or MM07 might have therapeutic potentials against the development of PUs.

## Introduction

Pressure ulcers (PUs) are one of the common skin diseases which usually occur elder people and patients with perceptual or movement disorder. In addition, the patients with PUs sometimes cause fatal outcome by local and systemic infections. There has long been considered that the pathogenesis of PUs was associated with tissue damage caused by external force and ischemia. However, there has been increasing evidence that cutaneous ischemia-reperfusion (I/R) is important in the pathogenesis of PUs^1–3^. I/R injury is defined as cellular injury caused by the reperfusion of blood to previously ischemic tissue^4,5^. It is described that reperfusion of blood into the hypoxic tissue triggers off adverse events, including thrombosis and capillary narrowing which lead to vasculopathy, infiltration of inflammatory cells, production of proinflammatory cytokines and the apoptosis of resident cells and necrosis of tissues^6^. I/R injury causes multiple diseases, such as vascular infarction or spasm of brain, heart, kidney and skin. Reactive oxygen species (ROS), such as H_2_O_2_ and NO, are also known as a key player that exacerbate the tissues damage caused by I/R injury^7^.

Apelin, an endogenous ligand of G protein-coulpled receptor APJ (putative receptor protein related to the angiotensin receptor AT1)^8,9^ has multiple functions in the regulation of cardiovascular hemostasis, angiogenesis and adipose tissue function via apelin/APJ signaling^10–13^. APJ is expressed ubiquitously, especially in endothelial cells and vascular smooth muscle cells^8,9,11,12^. Furthermore, recent studies demonstrated that apelin/APJ signaling prevented oxidative stress, resulting in the inhibition of diabetic microvascular complications^14,15^ and the protection of tissue damage caused by I/R injury in kidney, heart and brain^16–18^. However, it has not been elucidated whether apelin/APJ signaling regulates cutaneous I/R injury associated with PUs. Herein, we analyzed the role and mechanisms of the regulation of cutaneous I/R-induced PUs formation by apelin/APJ signaling.

## Results

### Expression of apelin during cutaneous I/R *in vivo*

**Figure 1:**
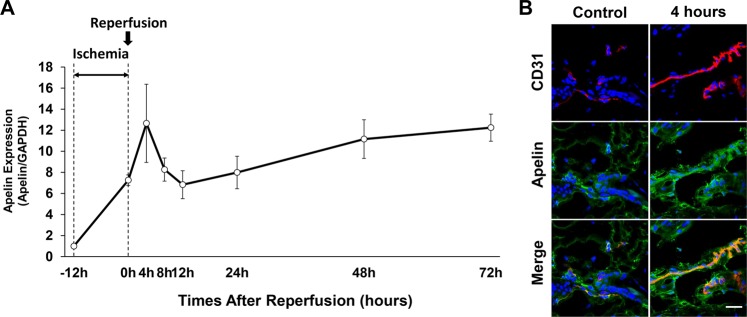
Apelin expression and distribution during ischemia-reperfusion (I/R) injury. (**A**) Quantification of apelin mRNA levels in the I/R site from the beginning of ischemia to 72 hours after reperfusion by quantitative reverse transcriptase–PCR (RT–PCR). The end of ischemia was assigned 0 hour. Data are relative to mRNA level in -12 hours. Values were determined in n = 4. (**B**) Expression and distribution of apelin in the skin before ischemia (control) and at 4 hours after reperfusion (red: CD31, green: apelin). Scale bar = 20 μm. All values represent mean ± SEM. **P < 0.01, *P < 0.05.

First, we investigated the apelin expression during cutaneous I/R injury. mRNA levels of apelin expression in the skin of I/R site were increased after ischemia (0 h) and 4 hours after reperfusion (Fig. 1A). Then, apelin expression was immediately decreased from 4 to 12 hours after reperfusion. After that, it was gradually increased following 1 to 3 days after I/R injury (Fig. 1A). To examine the amount and distribution of apelin under normal and after reperfusion condition in murine skin, immunofluorescence staining was performed. Apelin was accumulated around CD31^+^ endothelial cells in the dermis in the peripheral area of I/R site, and the expression of apelin was increased after reperfusion (Fig. 1B). These results suggest that apelin expression might be increased by cutaneous I/R-induced hypoxia, and apelin may be mainly produced by I/R affected endothelial cells in the peripheral area of I/R site.

### Apelin protected PUs formation after cutaneous I/R in mice model *in vivo*

**Figure 2:**
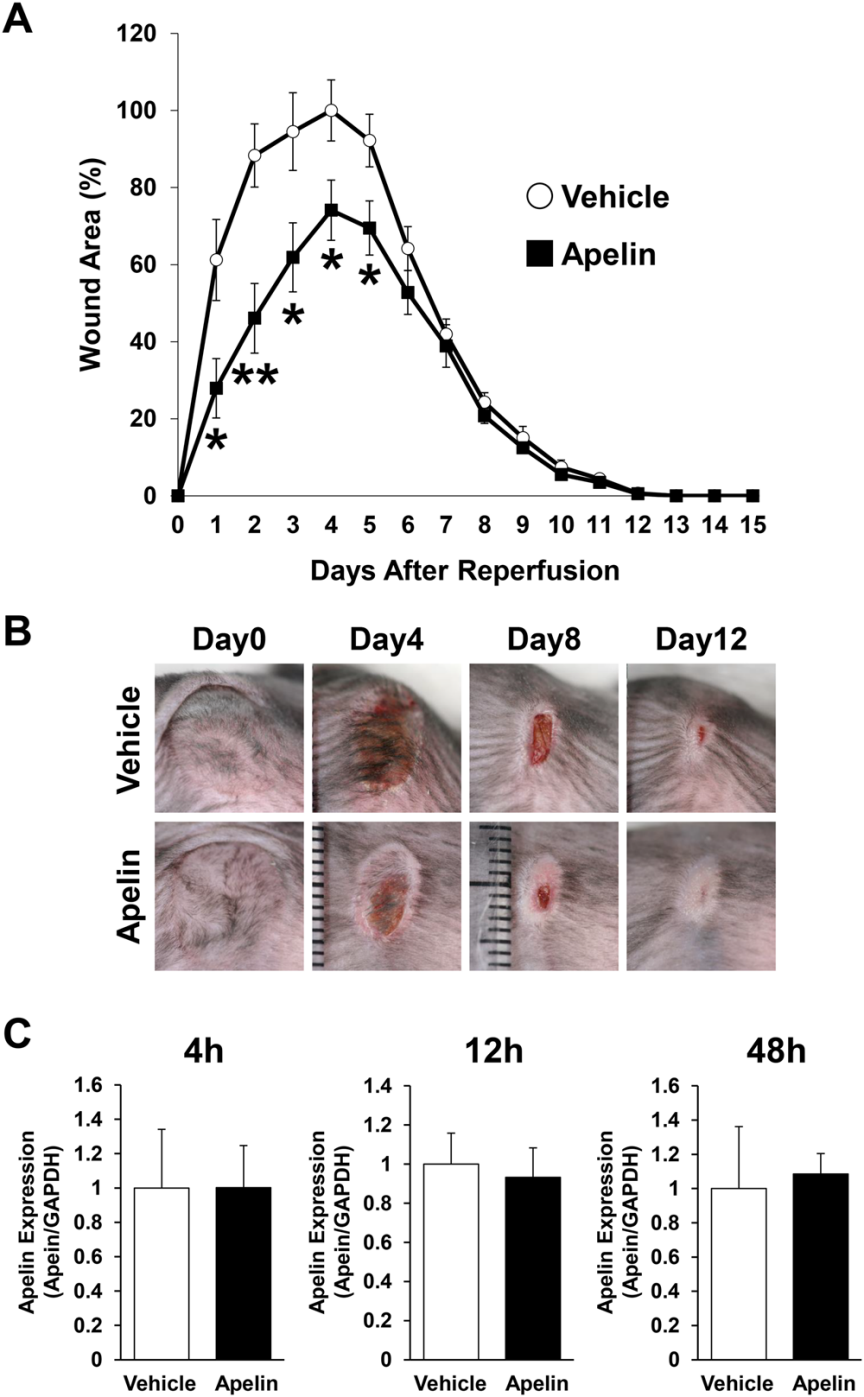
Apelin protected PUs formation in cutaneous I/R injury mouse model. (**A**) The size of the wound area after I/R injury in normal C57BL/6 mice treated with subcutaneous injection of apelin (10 ng/mice) or phosphate-buffered saline as a control. The size of the ulcer in control mice at 4 days after reperfusion was assigned a value of 100% (vehicle: n = 9, apelin: n = 10, for each time point and group). (**B**) Representative images of the wound after cutaneous I/R in control or apelin treated mice at 0, 4, 8, and 12 days after reperfusion. (**C**) Comparison of mRNA levels of apelin expression during wound healing in I/R site between control and apelin-treated mice at 4, 12 and 48 hours after I/R injury. n = 5. All values represent mean ± SEM. **P < 0.01, *P < 0.05.

To assess the effect of apelin ([Pyr^1^]-Apelin-13) on the development of PUs after cutaneous I/R *in vivo*, we compared wound area after I/R injury in normal C57BL/6 mice treated with subcutaneous injection of apelin or PBS as a control. We used a simple, reproducible and noninvasive experimental mouse model to evaluate the pathogenesis of cutaneous PUs by I/R *in vivo*^3,19^. Administration of apelin significantly inhibited the formation of PUs (Fig. 2A,B). The wound area in apelin-injected mice was significantly smaller that in control mice from 1 to 5 days after reperfusion. At 4 days after reperfusion, the size of wound area in the apelin-injected mice was 70% of that in the control mice. Next, we examined the expression of apelin in the wound healing process in I/R mice with or without apelin treatment, and found that administration of apelin did not change the mRNA levels of apelin in I/R site at 4, 12 and 48 hours after cutaneous I/R injury (Fig. 2C). These results demonstrate that apelin partially protected the formation of cutaneous PUs after cutaneous I/R.

### Injection of apelin protected vascular loss by cutaneous I/R injury

**Figure 3:**
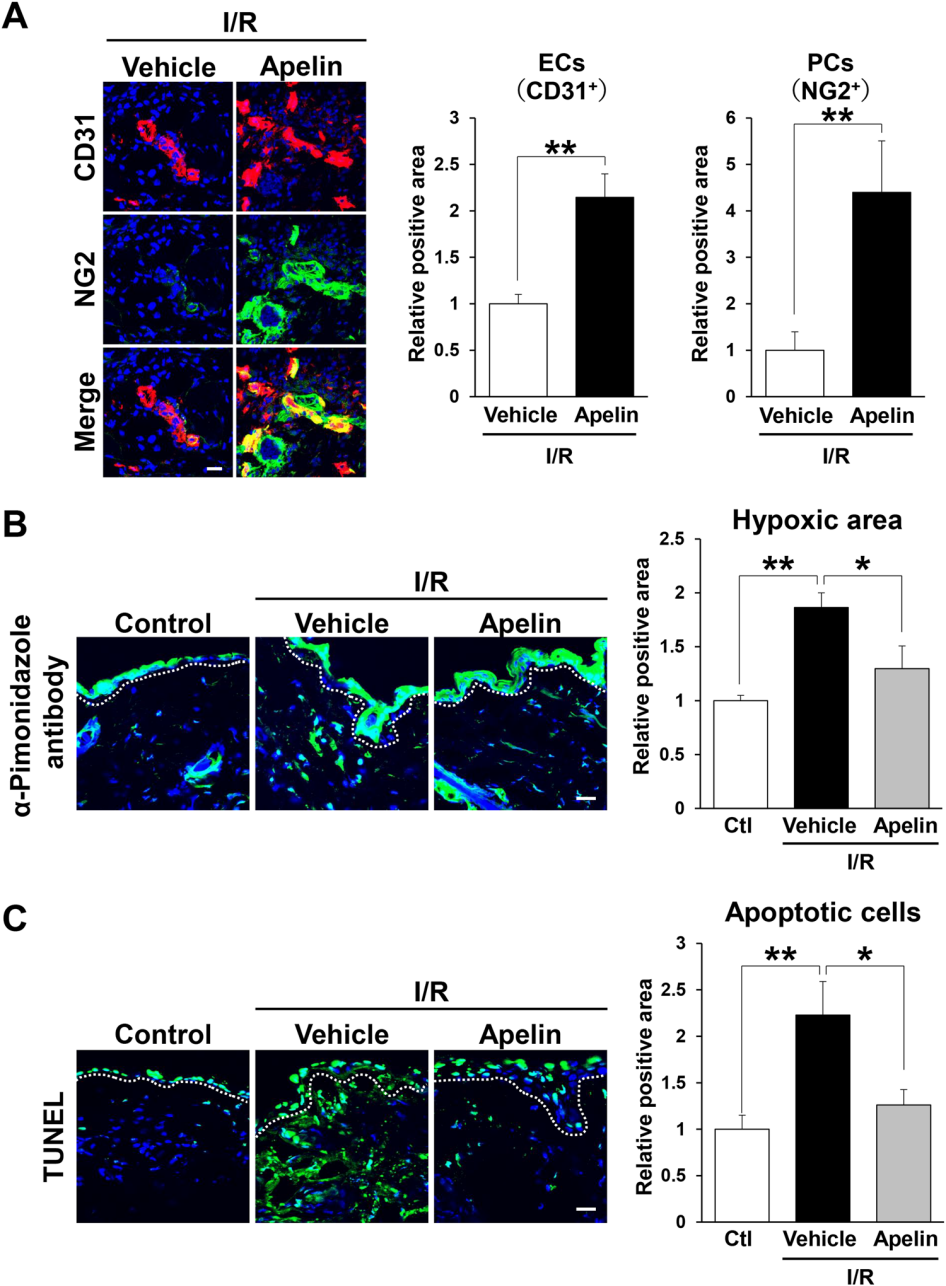
Apelin protected vascular reduction and suppressed hypoxia and apoptosis after cutaneous I/R. (**A**) The amount of CD31^+^ ECs and NG2^+^ pericytes in the cutaneous I/R area at 4 days after reperfusion. (**B**) The amount of pimonidazole^+^ hypoxic area in cutaneous I/R site at 1 day after reperfusion. Quantification of the pimonidazole^+^ areas in 6 random microscopic fields from the center of I/R area in n = 3 mice per groups was performed using ImageJ software. Positive area in control mice was assigned a value of 1. Scale bar = 20 μm. (**C**) The number of apoptotic cells in the I/R site at 1 day after reperfusion was determined by counting both TUNEL- and DAPI-positive cells. Values were determined in 6 random microscopic fields in n = 3 mice per group. All values represent mean ± SEM. **P < 0.01, *P < 0.05.

We previously identified that the number of blood vessels was reduced after cutaneous I/R injury in mice^20^. Therefore, we investigated the effect of apelin on vascular loss caused by cutaneous I/R injury. At 4 days after reperfusion, the numbers of CD31^+^ endothelial cells and NG2^+^ pericytes around I/R areas in apelin-treated mice were significantly higher than those in control mice (Fig. 3A). These results suggest that apelin might prevent the reduction of vascularity after cutaneous I/R injury.

### Apelin suppressed hypoxia and apoptosis by cutaneous I/R injury

We next examined the influence of apelin treatment on tissue hypoxia after cutaneous I/R injury in mice. Hypoxic area in the skin tissue was analyzed using the hypoxia marker pimonidazole. At one day after cutaneous I/R, hypoxic area in the I/R site was increased compared with control mice without I/R (Fig. 3B). However, hypoxia staining positive area in I/R site in apelin-treated mice were significantly decreased compared to those in vehicle-treated mice (Fig. 3B). As hypoxia activates cellular apoptosis^21^, we examined the influence of apelin on the number of apoptotic cells in I/R areas in mice. TUNEL staining revealed that administration of apelin into I/R site reduced the increased number of apoptotic cells after I/R injury (Fig. 3C). These results suggest that administration of apelin might suppress hypoxic area and apoptosis in I/R site after cutaneous I/R injury.

### Apelin regulated the infiltration of inflammatory cells and the expressions of cytokines and growth factors after cutaneous I/R

**Figure 4:**
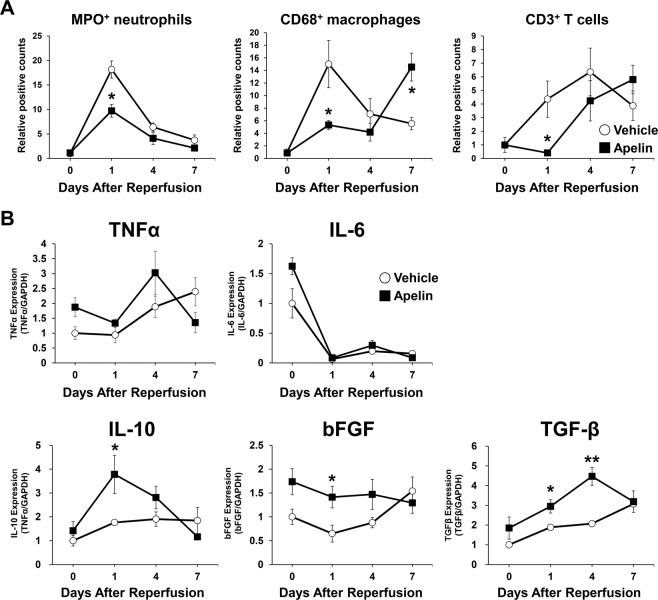
Apelin regulated the infiltration of inflammatory cells and the expressions of cytokines and growth factors after cutaneous I/R. (**A**) Quantification of the numbers of infiltrating MPO^+^ neutrophils, CD68^+^ macrophages and CD3^+^ T cells in I/R site at 0, 1, 4 and 7 days after I/R treated with or without apelin. Values were determined by counting cells in six random microscopic fields in n = 4–5 mice per groups. (**B**) mRNA levels of growth factors and cytokines in I/R site at 0, 1, 4 and 7 days after I/R treated with or without apelin. n = 4–5 mice in each group. The number of cells or mRNA levels in control mice were assigned values of 1. All values represent mean ± SEM. **P < 0.01, *P < 0.05.

We previously reported that cutaneous I/R injury induced the infiltration of inflammatory cells in I/R site, and the growth factors and cytokines produced by inflammatory cells were key factors following the development of PUs and wound healing process^22,23^. To examine the effect of apelin administration on the dynamics of infiltration of inflammatory cells (MPO^+^ neutrophils, CD68^+^ macrophages and CD3^+^ T cells), immunohistochemical studies were performed using skin tissue of I/R site between control and apelin-treated mice at day 0, 1, 4 and 7 after I/R injury. At day 1, the number of MPO^+^ neutrophils was significantly inhibited by apelin treatment (Fig. 4A). However, there were no significant difference between control and apelin-treated mice at day 0, 4 and 7. The numbers of CD68^+^ macrophages and CD3^+^ T cells at day1 was significantly inhibited by apelin, and the peak of the numbers of infiltrated CD68^+^ macrophages and CD3^+^ T cells were delayed by apelin treatment (Fig. 4A).

Furthermore, we examined the expression levels of growth factors and cytokines expression, including tumor necrosis factor-α (TNF-α), transforming growth factor-β (TGF-β), interleulin-6 (IL-6), IL-10 and basic fibroblast growth factor (bFGF) in I/R site through wound healing process by real-time PCR assay. The expression levels of proinflammatory cytokines (TNF-α, IL-6) were not different between control and apelin-treated mice through day 0 to 7. The mRNA levels of IL-10 and bFGF expressions were significantly increased at day 1 by apelin treatment, and TGF-β expressions was significantly increased at day 1 to 4 by apelin treatment (Fig. 4B). These results suggest that apelin might suppress recruitment of inflammatory cells at early phase of wound healing, and change the expressions of several growth factors and cytokines in the I/R site.

### Apelin suppressed oxidative stress after cutaneous I/R

**Figure 5:**
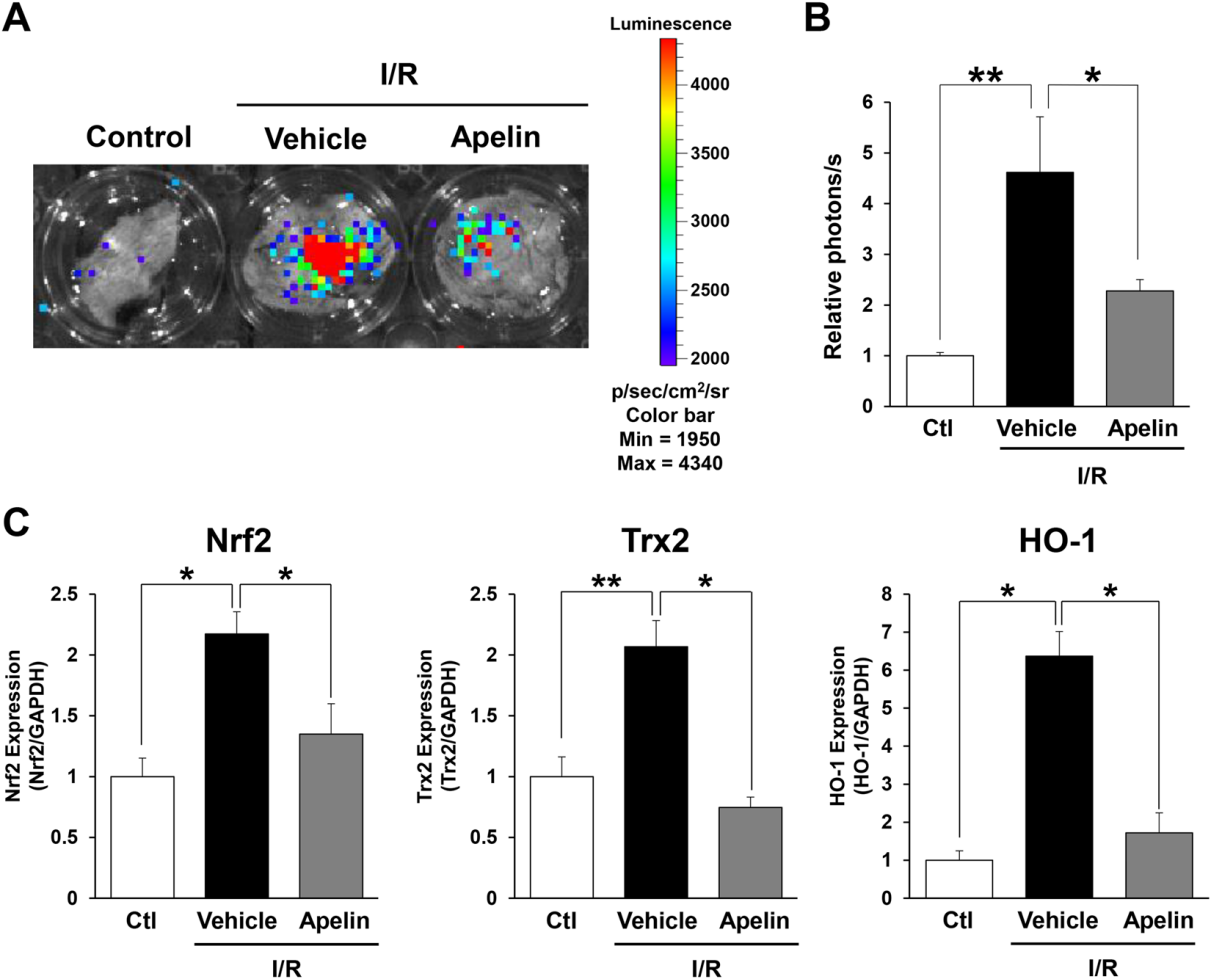
Apelin reduced oxidative stress induced by cutaneous I/R injury *in vivo*. (**A**) Representative image of luminescence signals in cutaneous I/R area in OKD48 mice at 24 hours after reperfusion (Day 1). The color scale bar shows the photon counts (photon (p)/sec/cm^2^/sr). (**B**) Quantification of luminescence signals in cutaneous I/R area in OKD 48 mice. n = 4 in each group. (**C**) mRNA levels of oxidative stress-associated factors, Nrf2, Trx2 and HO-1 in the I/R area at 24 hours after reperfusion (Day 1). mRNA levels in control mice were assigned as values of 1. Values represent mean ± SEM. n = 4–5 mice in each group. **P < 0.01, *P < 0.05.

Next, we investigated the effect of apelin on oxidative stress induced by I/R injury by using OKD48 (Keap1-dependent oxidative stress detector, NO-48) mice^24^. OKD48 mice have a transgene encoding a modified Nuclear factor erythroid 2-related factor 2 (Nrf2), which is an essential transcription factor for expression of antioxidative stress genes^25^. By using this mouse strain, oxidative stress *in vivo* can be detected with luminescence signals^20,24,26^. At 1 day after reperfusion, luminescence signal was significantly enhanced in the I/R area, and this signal was drastically reduced by administration of apelin (Fig. 5A,B). Additionally, the mRNA expression levels of oxidative stress-related factors, including Nrf2, heme oxygenase 1 (HO-1) and thioredoxin-2 (Trx2) in the I/R area were examined using real-time PCR. It has been reported that I/R injury enhances the expression of Nrf2, Trx2 and HO-1 in the brain and liver^27–29^. Consistent with previous results, mRNA levels of Nrf2, HO-1 and Trx2 expressions were significantly upregulated after cutaneous I/R injury, and apelin injection suppressed those gene expressions (Fig. 5C). These results suggest that the oxidative stress in cutaneous I/R area might be inhibited by apelin injection.

### Apelin suppressed ROS production and apoptosis driven by oxidative stress *in vitro*

**Figure 6:**
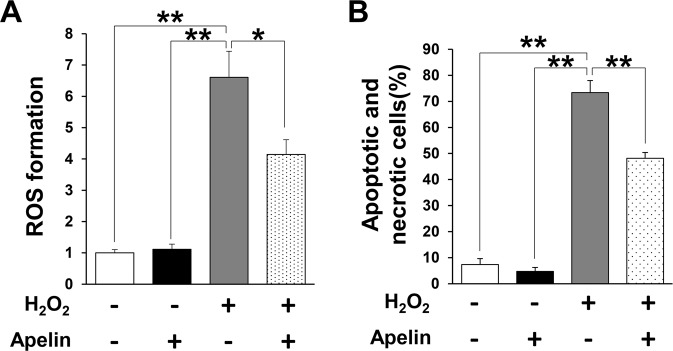
Apelin inhibited the oxidant-induced intracellular accumulation of ROS in endothelial cells and apoptosis in fibroblasts *in vitro*. (**A**) Quantification of H_2_O_2_-induced (0.75 mM) intracellular ROS production in HUVECs treated with or without apelin. ROS formation in cells without treatment was assigned as a value of 1. n = 4 in each group. (**B**) Representative data of the amount of early apoptotic cells (Annexin V^+^, 7-AAD^−^) and necrotic cells (Annexin V^+^, 7-AAD^+^) in NIH3T3 fibroblasts with or without H_2_O_2_ (0.75 mM) and/or apelin. (**C**) Quantitation of the amount of early apoptotic cells (Annexin V^+^, 7-AAD^−^) and necrotic cells (Annexin V^+^, 7-AAD^+^) in NIH3T3 fibroblasts with or without H_2_O_2_ (0.75 mM) and/or apelin. Values represent means ± SEM in four independent experiments. **P < 0.01, *P < 0.05.

Next, we examined whether apelin suppress ROS production and oxidative stress-induced apoptosis of endothelial cells and fibroblasts *in vitro*. H_2_O_2_-induced ROS productions in endothelial cells was suppressed by apelin treatment (Fig. 6A). In addition, apelin treatment significantly reduced the amount of H_2_O_2_-induced early apoptotic and necrotic cells in fibroblasts (Fig. 6B). These results suggest that apelin might reduce oxidative stress and oxidative stress-induced cell apoptosis *in vitro*.

### MM07 protected PUs formation after cutaneous I/R injury *in vivo*

**Figure 7:**
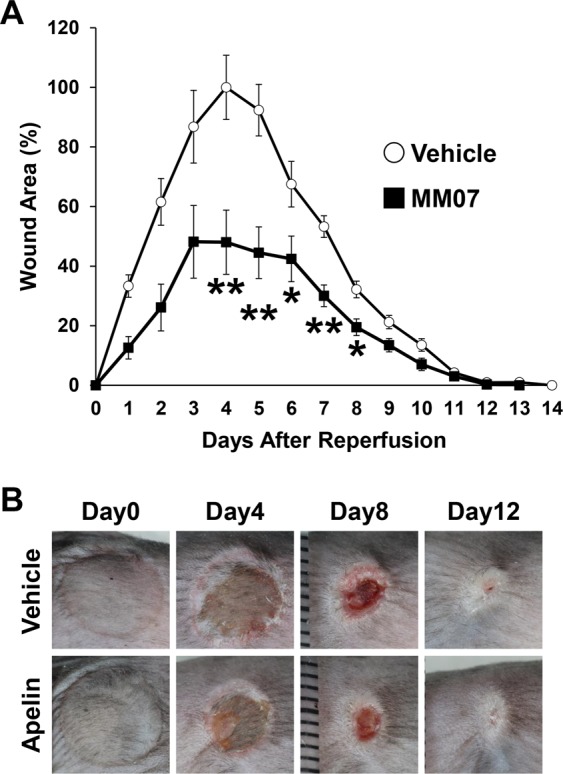
MM07 promoted angiogenesis around I/R area after cutaneous I/R. (**A**) The size of the wound area after I/R injury in normal C57BL/6 mice treated with subcutaneous injection of MM07 (1 ng/mice) or phosphate-buffered saline as a control. The size of the ulcer in control mice at 4 days after reperfusion was assigned a value of 100% (Control: n = 8–16, MM07: n = 8–16, for each time point and group). (**B**) Representative images of the wound after cutaneous I/R in control or apelin mice at 0, 4, 8, and 12 days after reperfusion. All values represent mean ± SEM. **P < 0.01, *P < 0.05.

Finally, we investigated the effect of the synthetic biased agonist of APJ, MM07^30,31^, on the development PUs formation after I/R injury *in vivo*. Since it has been reported that MM07 has greater potential than apelin to increase the forearm blood flow in human^30^, the amount of MM07 for treatment was designed 1/10 with respect to treatment with apelin (Fig. 2; 10 µg apelin, Fig. 7; 1 µg MM07). Wound area after I/R injury in C57BL/6 mice treated with subcutaneous injection of MM07 or PBS as a control were analyzed. Administration of MM07 significantly inhibited the formation of PUs after cutaneous I/R injury (Fig. 7A,B). At 4 days after reperfusion, the size of the wound area in the MM07-injected mice was 50% of that in the control mice. These results suggest that MM07 might have protective effects on the development of PUs formation after cutaneous I/R injury, and that MM07 might have greater potential than apelin to inhibit PUs formation *in vivo*.

## Discussion

At first, we investigated the expression of apelin during cutaneous I/R injury. We found that the expression of apelin was increased by ischemia, and then it was maximized at 4 hours after reperfusion and decreased until 12 hours. After that, it was gradually upregulated again in time dependent manner, suggesting that hypoxia and/or inflammatory pathways after I/R injury might induce the upregulation of apelin expression in I/R site.

Several studies reported that apelin has protective effect for tissue damage after I/R injury or traumatic injury via suppression of inflammatory cytokines, such as TNF-α, IL-1β and IL-6, oxidative stress, autophagy and N-methyl-D-aspartate (NMDA)-induced excitotoxicity^16,32,33^. However, the role of apelin in cutaneous I/R injury is unknown. Therefore, this is the first study to identify the mechanisms of protective regulation in cutaneous I/R injury by apelin/APJ signaling.

It has been reported that the number of blood vessels in I/R site were reduced after cutaneous I/R injury, indicating that hypoxia-induced ROS might cause cellular damage and apoptosis^7,20^. In this study, we assessed vascularity in I/R areas and identified that the reductions of CD31^+^ endothelial cells and NG2^+^ pericytes in I/R areas were inhibited by apelin administration, suggesting that apelin might inhibit the damage of blood vessels induced by cutaneous I/R. Apelin has been described to be an activator of angiogenesis and increase blood flow^30,34,35^, suggesting that administration of apelin might increase blood flow and angiogenesis, leading to the inhibition of I/R injury-induced vascular damages and hypoxic area in I/R site.

In addition, we demonstrated that enhanced oxidative stress caused by cutaneous I/R was reduced by injection of apelin in OKD48 mice. We also identified that oxidative stress related factors in I/R site were also suppressed in apelin-treated mice. Furthermore, we identified the anti-oxidative effects of apelin through the analysis of the production of ROS in endothelial cells and cellular apoptosis/necrosis in fibroblasts under oxidative stress *in vitro*. These results suggest that administration of apelin might suppress the oxidative stress and apoptosis via the inhibition of ROS production after cutaneous I/R injury. There are several studies regarding the mechanisms of the reduction of ROS production by apelin. For example, Zeng *et al*. reported that apelin suppressed ROS production and enhanced superoxide dismutase (SOD) activity in primary cultured myocardial cells after I/R injury^17^. SOD is an enzyme that degrades the ROS generated in cells by oxidative stress. Additionally, it has been reported that apelin-APJ signaling activates the AMP-activated protein kinase (AMPK)/Nrf2 signals, and MAP kinase pathway, resulting in the upregulation of anti-oxidant enzyme, including SOD-1 under oxidative stress condition in adipocytes and neuronal cells^36,37^. These mechanisms may be involved in the suppression of ROS production and oxidative stress by apelin in our study, however further investigation is required.

Resent study demonstrated that the proinflammatory cytokines derived from inflammatory cells, including neutrophils and macrophages, are key factors in the development of PUs^19,22,23^. In this study, we found the numbers of inflammatory cells were suppressed in apelin-treated mice at day 1. It has been reported that IL-10, bFGF and TGF-β prevent tissue damage after I/R injury^38–40^. We demonstrated that qPCR analysis revealed that apelin administration enhanced the expression of these tissue protective cytokines and growth factors, such as IL-10, bFGF and TGF-β in I/R site, however, further studies will be needed to clarify the precise mechanisms.

It has been known that chronic administration of apelin induces β-arrestin-mediated internalization of APJ and receptor desensitization, suggesting that there is a limitation for the clinical use of apelin^31^. On the other hand, MM07 is cyclic apelin peptide, and preferentially activates G protein responses with low potency in β-arrestin-mediated receptor internalization^30,31^. Consistent with these findings, it has been reported that intrabrachial infusion of MM07 had greater potential than apelin to increase blood flow in human^30^. Similar to previous results, we identified that inhibitory effect on the development of PUs by MM07 was higher than that by apelin.

Administration of apelin/MM07 suppressed the development of PUs in early phase, however there was no difference in the whole period of wound healing in our study. Since aperin/MM07 has a vasodilatory effect and an oxidative stress inhibitory effect, there is a possibility that it promotes wound healing. However, we considered that there was no difference in the whole period of wound healing because of the strong skin contraction that occurs at the end of wound healing in mice. The limit of this model is that it is highly influenced by the effect of ulcer contraction induced by the fibrosis. This is a so much difference compared by PUs in the humans. Further study will be needed to examine the effect of apelin/MM07 on wound healing.

Taken together, we demonstrate that apelin/APJ signaling suppresses the formation of PUs induced by cutaneous I/R injury by preventing the reduction of blood vessels and the protection of tissue damage through the inhibition of oxidative stress induced by I/R injury. Exogenous apelin or MM07 administration has possible therapeutic potential for cutaneous I/R injury-induced PUs.

## Methods

### Animals

All experiments were approved by the Gunma University Animal Care and Experimentation Committee. C57BL/6 mice were purchased from the SLC (Shizuoka, Japan). OKD48 (Keap1-dependent oxidative stress detector, NO-48) mice were kindly provided from Dr. T. Iwawaki (Department of Life Science, Kanazawa Medical University, Ishikawa, Japan). Eight- to 12-week-old mice were used for all experiments. Mice were bred and maintained in the Institute of Experimental Animal Research of Gunma University under specific pathogen-free conditions. Mice were handled in accordance with the animal care guidelines of Gunma University.

### Antibodies

Antibodies (Abs) and their sources were as follows: rat anti-mouse CD31 monoclonal Ab (mAb) (MEC13.3; BD Bioscience, San Jose, CA), rabbit anti-mouse NG2 polyclonal Ab (pAb) (Millipore, Billerica, MA), rabbit anti-mouse apelin antibody pAb(Santa Cruz Biotechnology). Alexa 488-, Alexa 568-conjugated secondary Abs were obtained from Invitrogen (Carlsbad, CA). HRP-conjugated goat anti-mouse or anti-rabbit secondary Abs were obtained from Dako (Glostrup, Denmark).

### I/R cycles and analysis

The I/R model that has been previously reported was used^2,3,19,20,22,23,26^. Briefly, mice were anesthetized, and hair was shaved and cleaned with 70% ethanol. The dorsal skin was gently pulled up and trapped between two round ferrite magnetic plates that had a 12-mm diameter (113 mm²) and 5 mm thick, with an average weight of 2.69 g and 1180 G magnetic forces (NeoMag Co, Ichikawa, Japan). Epidermis, dermis, subcutaneous fat layer and subcutaneous loose connective tissue layer, but not muscles, were pinched by magnetic plates. This process creates a compressive pressure of 50 mmHg between the two magnets^2,3^. It has been demonstrated that an external pressure of 50 mmHg is sufficient to induce skin necrosis and ulcer by reducing blood flow 80%^3^. Dorsal skin was trapped between magnetic palates for 12 hours, and then plates were removed. Mice were not immobilized, and not anesthetized during ischemia. All of the mice developed two round ulcers separated by a bridge of normal skin. To assess the effects of apelin ([[Pyr^1^]-Apelin-13) (Tocris) or MM07 (cyclo [1-6]CRPRLCHKGPMPF; synthesized by Sigma-Aldrich)^30,41^ on the development of PUs formation and wound healing after cutaneous I/R injury, 10 µg of apelin or 1 µg of MM07 per 200 µl phosphate buffered salts (PBS) or 200 µl saline as a control were injected into the dermis in the I/R site just after reperfusion. For analysis, each wound sites were digitally photographed at the indicated time points after wounding, and wound areas were measured on photographs using Image J (version1.48, NIH, Bethesda, MD).

### Histological examination and immunofluorescence staining

Immunofluorescence staining of frozen sections and analyses were performed as described previously^42^. Murine skins were removed and 4 μm frozen sections were prepared and fixed in 4% PFA in PBS for 30 minutes. After blocking with 3% dry milk-PBS supplemented with 5% normal donkey serum or 5% normal goat serum for 1 hour at room temperature, sections were stained with Abs of interest followed by Alexa 488-, Alexa 568-conjugated secondary Abs. Sections were counterstained with 4,6-diamidino-2-phenylindole (DAPI) to visualize nuclei, mounted in ProLong Gold antifade reagent (Life Technologies).

### Assessment of tissue hypoxia

Hypoxic areas after cutaneous I/R injury in I/R site were detected using the Hypoxyprobe-1 TM Omni kit (HPI, Burlington, MA) as previously described^20,43^. Pimonidazole HCl was injected by intraperitoneal (60 mg/kg) 30 min prior to sacrificing the mice. Murine skins were removed and 4μm frozen sections were prepared and fixed cold acetone (4 degrees Celsius) for 10 min. Sections were incubated overnight at 4 degrees Celsius with rabbit anti-pimonidazole antisera PAb2627 diluted 1:20 in PBS containing 0.1% bovine serum albumin and 0.1% Tween 20. Sections were incubated for 1 h Alexa 488-conjugated secondary Abs. Images (6 fields/section) were taken and visualized with a FV10i-DOC confocal laserscanning microscope (Olympus). The positive area was determined by ImageJ (version1.48, NIH, Bethesda, MD) in the field (x600).

### Cell cultures

Mouse embryonic fibroblast cells (NIH3T3) were kindly provided from Dr. S. Torii (Institute for Molecular and Cellular Regulation, Gunma University, Maebashi, Japan). Cells were maintained in Dulbecco’s modified Eagle’s medium (DMEM) containing 100 units/ml penicillin, 100 μg/ml streptomycin and 10% fetal calf serum (FCS). HUVEC were purchased from ATCC (Manassan, VA). HUVEC were maintained in EBM-2 basal medium (Lonza, Basel, Switzerland) supplemented with EGM-2 Single Quot Kit Suppl. & Growth Factors (Lonza).

### ROS detection assay *in vitro*

Cellular ROS production were detected as previously^26^. HUVECs (2.5 × 10^4^ cells) were cultured in (100 µl/well OptiPlate^TM^-96F microplate: Perkin Elmer) incubated at 37 degrees Celsius overnight. Cells were stimulated with 0.75 mM H_2_O_2_ (100 µl/well) 2 hours with or without [Pyr^1^]-Apelin-13 (100 nM). After that ROS levels was measured with DCFDA Cellular ROS Detection Assay Kit (abcam, Cambridge, UK) according to the manufacturer’s protocol with fluorescent microplate measurement.

### Apoptosis and necrosis analysis with flow cytometry

Flow cytometric analysis of apoptosis was performed as described previously^20,44^. NIH3T3 cells were incubated in control medium or apelin with or without H_2_O_2_ (0.75 mM) for 24 hours before apoptosis analysis through flow cytometry. Both attached and nonattached cells in the supernatant were corrected. Cells were treated with fluorescein isothiocyanate (FITC)-conjugated Annexin V (BD Bioscience) and 7-amino-actinomycinD (7-AAD), and analyzed with an Attune Focusing Cytometer (Thermo Fisher Scientific, Waltham, MA, USA). Data were processed using FlowJo software (Tree Star Inc., Ashland, OR, USA). Cells that stained positive for Annexin V and negative for 7-AAD were considered to be early apoptotic cells.

### Real-time RT-PCR

To analyse the level of mRNA expression at the I/R site by real-time RT-PCR, whole skin samples at the I/R site were used. Total RNA was isolated using an RNeasy Mini Kit (Qiagen, Valencia, CA) and was subjected to reverse transcription using a GoScript Reverse Transcription System for RT-PCR (Promega) according to the manufacturer’s instructions. Quantitative RT-PCR was performed with the SYBR system (Applied Biosystems, Foster City, CA) using ABI 7300 real-time PCR instrumentation (Life Technologies) according to the manufacturer’s instructions. SYBR probes and primers for Apelin, Nrf2, Trx2, HO-1 and Glyceraldehyde-3-phosphate dehydrogenase (GAPDH) were purchased from Sigma (St. Louis, MO) and Takara Bio Inc. (Otsu, Japan). As an internal control, the levels of GAPDH mRNA were quantified in parallel with the target genes. Normalization and fold-changes were calculated using the comparative Ct method.

### Statistics

*P* values were calculated using the Student’s *t*-test (two-sided) or by analysis of one-way ANOVA followed by Bonferroni’s post test as appropriate. Error bars represent standard errors of the mean, and numbers of experiments (n) are as indicated.
